# Lymphopenia-Induced Proliferation in Aire-Deficient Mice Helps to Explain Their Autoimmunity and Differences from Human Patients

**DOI:** 10.3389/fimmu.2014.00051

**Published:** 2014-02-13

**Authors:** Kai Kisand, Pärt Peterson, Martti Laan

**Affiliations:** ^1^Molecular Pathology, Institute of Biomedicine and Translational Medicine, University of Tartu, Tartu, Estonia

**Keywords:** AIRE, APECED, lymphopenia-induced proliferation, thymus, negative selection, autoantigens, immune privilege, NOD

## Abstract

Studies on autoimmune polyendocrinopathy candidiasis ectodermal dystrophy (APECED) and its mouse model – both caused by mutant *AIRE* – have greatly advanced the understanding of thymic processes that generate a self-tolerant T-cell repertoire. Much is now known about the molecular mechanisms by which AIRE induces tissue-specific antigen expression in thymic epithelium, and how this leads to negative selection of auto-reactive thymocytes. However, we still do not understand the processes that lead to the activation of any infrequent naïve auto-reactive T-cells exported by AIRE-deficient thymi. Also, the striking phenotypic differences between APECED and its mouse models have puzzled researchers for years. The aim of this review is to suggest explanations for some of these unanswered questions, based on a fresh view of published experiments. We review evidence that auto-reactive T-cells can be activated by the prolonged neonatal lymphopenia that naturally develops in young Aire-deficient mice due to delayed export of mature thymocytes. Lymphopenia-induced proliferation (LIP) helps to fill the empty space; by favoring auto-reactive T-cells, it also leads to lymphocyte infiltration in the same tissues as in day 3 thymectomized animals. The LIP becomes uncontrolled when loss of Aire is combined with defects in genes responsible for anergy induction and Treg responsiveness, or in signaling from the T-cell receptor and homeostatic cytokines. In APECED patients, LIP is much less likely to be involved in activation of naïve auto-reactive T-cells, as humans are born with a more mature immune system than in neonatal mice. We suggest that human AIRE-deficiency presents with different phenotypes because of additional precipitating factors that compound the defective negative selection of potentially autoaggressive tissue-specific thymocytes.

## Introduction

The autoimmune regulator (AIRE) is a transcriptional activator with a restricted expression pattern and important functions in medullary thymic epithelial cells (mTECs) (1). The thymus is the organ where a self-tolerant T-cell repertoire is established via positive and negative selection of thymocytes. To ensure tolerance toward the set of tissue-specific antigens (TSAs) from different peripheral organs, mTECs “promiscuously” express thousands of TSAs that are then presented to developing thymocytes; one of the best known among them is insulin (2, 3). AIRE is the best characterized transcriptional regulator in mTECs. It is generally accepted that its main thymic role is to ensure negative selection of thymocytes with T-cell receptors (TCRs) with high affinities for epitopes from TSAs. At first sight, this idea seems to fit with the variety of endocrine, ectodermal, and lymphoid autoimmune diseases that present in patients with *AIRE* mutations and comprise the Autoimmune polyendocrinopathy candidiasis ectodermal dystrophy (APECED) or autoimmune polyendocrine syndrome type I (APS-I) syndrome (4–6). However, there is curiously little discussion about how these infrequent naïve auto-reactive T-cells that escape negative selection in AIRE-deficient thymi are activated to cause disease in the periphery, or about the rather consistent early onset of its highly unusual cardinal manifestations, or about the strikingly different phenotypes in *Aire*^−/−^ mice (7–9). Table 1 lists the autoimmune features of AIRE-deficient humans vs. mice and highlights their surprisingly limited overlap (7–21). Here, we propose the hypotheses that defective thymic negative selection is not sufficient by itself to induce autoimmunity and that these differences in disease phenotypes reflect distinct varieties of additional influences in *Aire*^−/−^ mice vs. humans.

**Table 1:** **Phenotypes and autoantibodies differ between APECED patients and *Aire*^−/^^−^ mice**.

APECED patients^a^	*Aire*^−/−^mice^b^
**DISEASES/IMMUNE CELL INFILTRATIONS**	
Chronic mucocutaneous candidiasis
Hypoparathyroidism
Addison’s disease
**Ovarian failure**	**Infertility**
**Testicular failure**
Hypopituitarism
Autoimmune hepatitis	Liver infiltration
Intestinal dysfunction
Pancreatitis
Tubulointerstitial nephritis
Interstitial lung disease	Lung infiltration
Alopecia
Vitiligo
Rash with fever
Asplenia
Keratoconjunctivitis
Dental enamel dysplasia
Nail dystrophy
Type 1 diabetes
Hypothyroidism
CIPD (10)
Pernicious anemia	Gastritis
	Uveoretinitis
	Dacryoadenitis
	Salivary gland infiltration
**AUTOANTIBODIES TO:**	
Type I IFNs	
IL-22, IL-17F, **IL-17A**	**IL-17A** (IL-17F) (11)
NALP5	
CaSR	
P450c17, P450c21, P450scc	
IA-2, GAD65	
TG, TPO	
TDRD6	
AADC	
P450 1A2	
TPH	
HDC	
TH	
SOX9/SOX10	
KCNRG	
Myelin protein zero (12)	
LPLUNC1 (13)	Vomeromodulin (13)
**BPIFB1** (14)	**BPIFB9** (14)
	OBP1a (16)
	SVS2 (17)
	IRBP (15)
	alpha-fodrin (18)
	TRP-1 (19)
	Mucin 6 (20)

*^a^Autoimmune phenotypes of APECED patients and their autoantibody reactivities are summarized from (21)*.

*^b^Summarized from (9), only *Aire*^−/−^ mice on C57BL/6 and BALBc backgrounds without additional immune defects are included*.

*CIDP, Chronic inflammatory demyelinating polyneuropathy; NALP5, NACHT leucine-rich-repeat protein 5; CaSR, calcium-sensing receptor; P450c17, steroid 17-α-hydroxylase; P450c21, steroid 21-hydroxylase; P450scc, side chain cleavage enzyme; IA-2, islet antigen-2; GAD65, glutamic acid decarboxylase; TG, thyroglobulin; TPO, thyroid peroxidase; TDRD6, tudor domain containing protein 6; AADC, aromatic l-amino acid decarboxylase; P450 1A2, cytochrome P450 1A2; TPH, tryptophan hydroxylase; HDC, histidine decarboxylase; TH, tyrosine hydroxylase; KCNRG, potassium channel-regulating protein; BPIFB1, 1 bactericidal/permeability-increasing fold-containing B1; OBP1a, odorant binding protein 1a; SVS2, seminal vesicle secretory protein 2; IRBP, interphotoreceptor retinoid-binding protein; TRP-1, tyrosinase-related protein-1; LPLUNC1, Long palate lung nasal epithelium clone*.

*Shared autoimmune features are indicated in bold*.

## AIRE is Responsible for Negative Selection of TSA-Specific Thymocytes

The normal roles of Aire in TSA up-regulation by mTECs, and thus in central tolerance induction, are firmly established. In mice transgenic for single TCRs specific for immune-dominant epitopes from hen egg lysozyme (HEL) or ovalbumin (OVA), large proportions of thymocytes are efficiently deleted if their neo-self-antigens are expressed under Aire-dependent gene promoters. Membrane-bound HEL or OVA (mHEL or mOVA) under the rat insulin promoter (RIP) is expressed in both pancreatic β cells and the thymus (22, 23), and mHEL under the interphotoreceptor retinoid-binding protein (IRBP) promoter in both retina and thymus (24). When these mice are crossed with the respective TCR-transgenic animals, their clonotypic thymocytes are deleted with 75–97% efficiency, but only in mice with intact Aire, highlighting its indispensable role in negative selection. Moreover, the prevalence of neo-self-antigen-reactive T-cells is reduced still further in the periphery, underlining the importance of active peripheral tolerance mechanisms.

Interestingly, expression levels of the transgenes in the thymus varied in different studies. In a retinal neo-self-antigen model, the transgenic mRNA (*Escherichia coli* β-galactosidase under arrestin promoter) was undetectable even in the wild-type (wt) thymus (25). Whereas mHEL showed the expected Aire-dependent pattern of higher expression in wt than *Aire*^−/−^ mTECs (24, 26) (when driven by the insulin or IRBP promoters), transcript levels for RIP-driven mOVA were not markedly decreased in *Aire*^−/−^ thymi (22). This raises the possibility that, besides up-regulation of TSAs in the thymus Aire plays additional roles in generating self-tolerance, e.g., inducing the maturation of mTECs, as reviewed recently (27, 28). Loss of Aire also alters thymic architecture and mTEC ultrastructure (29, 30), and these effects reach back even to the immature Aire-negative mTEC subset (31). Indeed, there are reports that Aire-deficiency leads to breakdown of tolerance even to apparently Aire-independent antigens (18). Moreover, the development of the most mature single CD4 positive thymocyte subpopulation (CD69^−^, Qa-2^+^) is impaired in Aire-deficient thymi (32).

The role of Aire in negative selection has also been studied in TCR-transgenic models where clonotypic T-cells are targeted toward naturally expressed self-antigens such as the melanocyte-/melanoma-specific tyrosinase-related protein-1 (TRP-1). In these mice (on a *Rag*^−/−^ background), negative selection again depended on Aire; when its only change was the dominant negative *Aire* G228W point mutation, melanoma growth was decreased. Surprisingly, however, vitiligo was not reported in this study, although TRP-1 is also expressed in normal melanocytes (19).

The role of Aire in negative selection has also been studied in another TCR-transgenic model with reactivity to the major retinal autoantigen – IRBP. Although its thymic expression is reportedly Aire-dependent, clonotypic thymocytes were not deleted in any of three transgenic mouse lines on the uveitis-susceptible B10.RIII background (33). On the contrary, in two of them, the majority of CD4 single positive thymic T-cells bound IRBP–MHC dimers; strikingly they were several-fold more frequent than in wt animals (33). Uveitis developed spontaneously in these two mouse lines, but not in the third, where frequencies were lowest in both thymus and periphery: 6 and 1% respectively; those were still much higher than in *Aire*^−/−^ mice with no TCR-transgene (34). Clonotypic T-cell deletion was also incomplete in mice transgenic for an insulin B chain epitope-specific TCR, only a fraction of which developed diabetes (35).

Several studies have confirmed the importance of thymic negative selection of auto-reactive T-cells in physiological settings, i.e., in mice with un-manipulated T-cell repertoires (34, 36). Indeed, thymic stromal or lymphoid cells were necessary to confer tolerance to the central nervous system (CNS) antigen myelin proteolipid protein (PLP) (36). Importantly, susceptibility to experimental autoimmune encephalomyelitis (EAE) in SJL/J mice could be explained by the exclusion of the immunodominant epitope of PLP (for this strain) from the thymic isoform of PLP, and the export of potentially auto-reactive cells to the periphery (36). However, this model of EAE in SJL/J mice does not develop spontaneously, but requires immunization with antigen emulsified in complete Freund’s adjuvant (CFA).

## Naïve Auto-Reactive T-Cells Do Not Cause Autoimmunity by Default

According to current models, AIRE’s main role is to ensure negative selection of TSA-specific thymocytes. If so, self-reactive T-cells escaping from *Aire*^−/−^ thymi must normally be naïve and infrequent. Even when frequencies are much higher in TCR-transgenic models, disease penetrance is not always 100%, especially when the TCRs are expressed in CD4+ T-cells. In the TCR–TrpHEL model, with neoantigen expression in melanocytes, 12% of the animals remained free of vitiligo (37); in an RIP–OVA OTII model with neo-self-antigen expression in pancreatic β-cells, about 1/3 were persistently non-diabetic (23) in spite of large numbers of auto-reactive T-cells in the periphery. TSA-specific T-cells are much less frequent in *Aire*^−/−^ animals with un-manipulated T-cell repertoires. How their uncommon naïve thymic emigrants are activated to induce autoimmune disease in the periphery remains unexplained, one might expect them to get tolerized instead (38, 39). Indeed, when naïve T-cells encounter self-antigen in tissue-draining lymph nodes or spleen in wt mice, they undergo an initial burst of proliferation that is followed by deletion and anergy (40–44) or acquisition of regulatory T-cell (Treg) phenotypes (35, 45). In intriguing contrast, autoimmunity readily develops when naïve auto-reactive T-cells are transferred to lymphopenic hosts (46, 47).

## Lymphopenia Triggers Autoimmunity in *Aire*^−/−^ Mice

The striking similarities in manifestations in *Aire*^−/−^ and day 3 thymectomized mice (d3tx) have been noticed earlier (48–50). Both models show inflammatory infiltrates in similar tissues plus autoantibodies against some of their antigens in: stomach, thyroid, ovaries, prostate, pancreas, lacrimal and salivary glands, and testis (9, 18, 50–55). With both types of models, the manifestations even follow the same strain-specific preferences: e.g., generally lower autoimmune susceptibility in C57BL/6 mice, whereas gastritis is the most prevalent feature on the BALBc background.

In d3tx mice, the autoimmunity is explained by prolonged lymphopenia-induced proliferation (LIP) of auto-reactive lymphocytes that out-compete Tregs in susceptible animals (56, 57). Although normal neonatal mice show a physiologic lymphopenia, it does not induce substantial LIP (56). We have shown that, besides inducing TSA expression, thymic Aire normally upregulates several chemokines, especially CCR7 and CCR4 ligands, that attract immature thymocytes to the medulla. Their cortico-medullary migration is delayed in *Aire*^−/−^ mice, and that, in turn, delays the export of their mature progeny, prolonging the post-natal lymphopenia at least through day 5 (31). Interestingly, mice deficient in CCR7 (or its ligands) show not only similar delays in T-cell emigration from the thymus but also inflammatory infiltrates in the very organs listed above (58–60). We therefore hypothesize that LIP also contributes to these inflammatory infiltrates and compensates for the relatively low numbers of naïve auto-reactive T-cells that escape from *Aire*^−^*^/^*^−^thymi. This notion is supported by the evidence that the lymphopenia in irradiated *Aire*^−/−^mice increases the gastric autoimmunity (20); and that Aire expression is required only in the fetal and early post-natal periods to prevent autoimmunity (48).

Lymphopenia-induced proliferation is sometimes classified according to the rate of division of T-cells to homeostatic and spontaneous proliferation (56). It is highest when chronically lymphopenic adult mice are reconstituted with low numbers of lymphocytes (56, 61). In this case, T-cells respond to antigens derived from commensals, which probably translocate from the gut to lymphoid organs due to the host immunodeficiency (61). Commensals seem unlikely contributors to the LIP that occurs early in life, e.g., in d3tx mice. Nevertheless, LIP favors auto-reactive cells, as they get stronger signals through their TCRs as well as from homeostatic cytokines (IL-7 and IL-15) that are upregulated in lymphopenic hosts. As they concomitantly differentiate, these T-cells acquire the markers of activated memory cells (CD44^+^CD62L^−^) (62–66).

There are several indications of homeostatically proliferating T-cells in *Aire*^−/−^ mice, including signs of oligoclonality (67). Whereas thymocytes from Aire-deficient and wt mice showed no differences in TCR Vβ-chain CDR3 length and spectratype, splenic T-cells from *Aire*^−/−^ mice showed a clear alteration in the TCR repertoire distribution in 3 out of 24 Vβ families at 2 and 6 months of age (67). A more recent study also found slight perturbations in CDR3 Vβ length distribution, and significantly higher percentages of CD44+ T helper cells in spleens and lymph nodes of *Aire*^−/−^ mice than in wt controls (9). CD44 up-regulation in T-cells from *Aire*^−/−^ mice was also noted by Anderson et al. (68).

Looking for further activation of auto-reactive cells in lymphopenic conditions, Kekalainen et al. (69) transferred lymph node cells from *Aire*^+^ and *Aire*^−/−^ mice to immunodeficient hosts. However, although especially the CD8+ *Aire*^−/−^ T-cells proliferated more, there was no clinical disease, and the mild infiltrates in the livers, salivary glands, and pancreata did not differ from those in the controls. The rare auto-reactive cells in these animals had probably already been tolerized by peripheral mechanisms in the donors themselves. This suggests that prolonged lymphopenia in the neonatal period, together with export of naïve cells to the periphery, contributes substantially (but not exclusively) to the development of inflammatory infiltrates in *Aire*^−/−^ mice, and that the auto-reactive cells are subject to regulation in the periphery that prevents serious damage to the target organs.

Certain TCR-transgenic T-cells are also prone to homeostatic proliferation. These include the MHC-class I-restricted OT-I line recognizing a peptide from OVA (62). Interestingly, spontaneous diabetes already appears in neonatal RIP–OVA *Aire*^−/−^ OT-I mice (22). This severe autoimmunity might well have been potentiated by perinatal activation of the transgenic T-cells in these lymphopenic hosts.

## AIRE and LIP in Autoimmunity Against Privileged Organs

Autoantigens from some organs like the CNS/retina were thought to be sequestered from the immune system, which might therefore not be fully tolerant to them. It has been suggested that AIRE might play especially important roles in protecting these organs from autoimmune attack, e.g., provoked by local infections (49). Indeed, central deletion of auto-reactive thymocytes would be a particular priority for CNS and eye antigens, as regeneration is minimal in these tissues, and their peripheral tolerizing mechanisms might be inefficient. The intraocular compartments are isolated from the circulation – by barriers formed by tight junctions between the endothelial cells of the ciliary blood vessels, and between the lining epithelial cells; also in the retinal pigment epithelium (RPE) and the local endothelium (70–72). These barriers are impermeable to circulating soluble macromolecules and most cell types except for activated T-cells and immature antigen-presenting cells (APCs). In the other direction, any soluble retinal antigens (such as IRBP) shed physiologically or injected experimentally can drain via the aqueous fluid and episcleral veins to reach the thymus, liver, and spleen (70). The resulting systemic tolerance is termed anterior chamber-associated immune deviation (ACAID). The presumed privilege of the eye used to be attributed to paucity of APCs and lymphatics, but it is now known that there are rich networks of APCs and a functioning lymphatic system draining all parts of the eye, except the retina proper, via the submandibular node (70–72). Thus, ocular privilege is not due to a passive barrier, but instead depends on inducible active processes that can be transferred by immune cells.

One prominent feature in *Aire*^−/−^ mice is their retinal disease. Although it is extremely rare in APECED patients who frequently suffer from keratito conjunctivitis (4, 73), it affects ~30% of these mice by age 20 weeks on a C57BL/6 background (34). Recently, they were backcrossed onto the autoimmune uveitis-susceptible B10.RIII background to monitor eye pathology more carefully (74). Surprisingly, the spontaneous disease was milder on the *Aire*^−/−^ background than in the other two models (induced by immunization with IRBP + CFA or arising spontaneously in IRBP TCR-transgenic mice), and rarely caused blindness. Instead, it presented with relatively low-grade but multi-focal retinal inflammation and severe choroiditis, possibly hinting at moderately potent regulatory mechanisms.

There are many indications that EAU is enhanced by LIP of self-reactive T-cells (33, 75, 76). In intact wt recipients, IRBP-transgenic T-cells only induced uveitis after antigen-activation: recipients of naïve cells, even from the highest transgenic TCR-expressing line, remained disease-free. In telling contrast, naïve T-cells did induce disease when transferred to lymphopenic *Rag2*^−/−^ recipients, again implicating LIP in converting them into effector cells (33). In the same study, LIP was evidenced in the mouse lines with higher prevalences of TCR-transgenic T-cells by increases in CD44^+^CD62L^−^ activated T-cells, even in peripheral lymph nodes that do not drain the eye. This implicates LIP in these transgenic animals too, possibly due to aberrant thymic development, and probably lymphopenic periods earlier in life (33). LIP has also been identified as a potent activator of EAU in another transgenic model (76) and, interestingly, uveoretinitis develops in unimmunized d3tx mice if subsequently injected with anti-CD25 to deplete CD25^+^CD4^+^ Tregs (75).

## Reversal of Lymphopenia Alleviates Autoimmunity

Autoimmunity that results from LIP should be down-modulated by transfer of lymphocytes. This indeed occurs in *Aire*^−/−^ mice, where the appearance of inflammatory infiltrates could be suppressed by introducing a controlled excess of T-cells from normal donors – by co-transplanting 1:4 mixes either of *Aire*^−/−^: wt stroma from thymic lobes, or of splenocytes, into athymic or *Rag*^−/−^recipients, respectively (22).

As the phenotypes of *Aire*^−/−^ mice are so mild, it is difficult to dissect the mechanisms that might be modulating their autoimmunity. Therefore, crosses of *Aire*^−/−^ with NOD mice have been used, as they develop earlier and more severe autoimmunity (48). In these crosses, Aire expression is especially important during perinatal life. Moreover, intraperitoneal injection of adult T-cells on days 1 and 7 conferred significant but not complete protection from this exaggerated autoimmunity (48) (see below).

## Is Absence of Self-Antigen from the Thymus Sufficient by Itself to Induce Organ-Specific Autoimmune Disease?

It is sometimes assumed that the autoimmunity results solely from the absence of a single autoantigen from the thymus in the presence of wt Aire. That is apparently contradicted by our hypothesis that prolonged lymphopenia in *Aire*^−/−^ mice is an important cofactor for auto-aggression, so we now discuss two models that might help to distinguish between these possibilities.

DeVoss et al. identified IRBP as the major target in autoimmune uveitis in *Aire*^−/−^ mice (15). Its thymic expression is Aire-dependent, although it is barely detectable in wt thymic stroma. Absence of IRBP in the thymic compartment alone was sufficient to cause disease when athymic nude mice were transplanted with fetal thymic stroma from IRBP^−/−^ mice or wt mice. Mononuclear infiltrates appeared in their retinae, but not in recipients of wt stroma. Here again, lymphopenia must have been an important early contributor, as the first thymic emigrants appeared to abnormal lymphopenic adults.

When DeVoss et al. also crossed *Aire*^−/−^ with IRBP^−/−^ mice, the retinae showed no infiltrates, as expected because there was no target for the IRBP-specific cells to attack. However, IRBP is secreted, and even reaches the vitreous, and eventually drains to the spleen and lymph nodes (77). Hence this major eye retinal autoantigen was missing from the peripheral immune system too, and was not available to fuel homeostatic proliferation of IRBP-specific T-cells. Also the IRBP^−/−^ retina is atrophic and might be depleted of other autoantigens.

Interestingly, when mice transgenic for mHEL under the IRBP promoter were crossed with HEL-specific TCR-transgenic mice, they showed severe spontaneous EAU even on a wt *Aire* background (24). Negative selection of clonotypic T-cells was not complete in this model, and many neo-self-antigen-specific T-cells were exported to the periphery. The mHEL – unlike soluble IRBP itself – may have failed to access lymphoid organs/induce peripheral tolerance. The resulting disease was already so severe that any exacerbating effect of Aire-deficiency was not detectable. If these HEL-specific clonotypic T-cells were susceptible to LIP due to cross-reactivity with some self epitopes (which has not been checked), that might well have contributed too.

In another study, mice were engineered specifically to prevent any insulin expression in mTECs, and to use only one of the two insulin genes (*Ins2*) in their pancreatic β-cells (78). They developed spontaneous diabetes within 3 weeks after birth. However, there are also some caveats with this study (79). The diabetes was not transferrable to immunodeficient adult hosts with lymphocytes or thymi from the transgenic mice, which showed only moderate insulitis (80). This apparently implicates the additionally impaired physiology of *Ins1*^−/−^ β-cells (compensatory hyperplasia, increased death during the developmental wave of apoptosis that occurs in normal development) in disease initiation in very young mice (81). In this model again, loss of thymic negative selection alone was not sufficient to cause clinical disease. Furthermore, since insulin is already secreted in the fetus, it should normally be available for thymic deletion, e.g., when presented by medullary dendritic cells, without promiscuous expression in mTECs, but its levels may be decreased prenatally in *Ins1*^−/−^ mice, reducing its availability for negative selection.

## Aire-Deficiency Becomes Lethal if Peripheral Back-Up Mechanisms are Eliminated

Two highly informative crosses of *Aire*^−/−^ mice – with strains with other immune defects – underline the importance of back-up mechanisms that are apparently responsible for the mildness of the disease phenotypes in *Aire*^−/−^ mice. Crosses onto *Cbl-b*-deficient or diabetes-prone NOD backgrounds show astonishing similarities (39, 53, 82). They both suffer from early wasting disease and succumb to acute exocrine pancreatitis around 3–4 weeks of age. *Aire*^−/−^/*Cbl-b*^−/−^ mice showed additional lymphocytic infiltrates in submandibular salivary glands and stomach (39), while Aire-deficiency on the NOD background was accompanied by severe pulmonitis and infiltrates in liver, salivary gland, prostate, ovary, stomach, and thyroid (53, 82).

Interestingly, mice deficient in Cbl-b alone are healthy in the absence of additional triggers (83), so it was a major surprise that crossing with *Aire*^−/−^ mice led to such severe disease. Cbl-b normally renders naïve T-cells highly dependent on co-stimulation; when it is deleted, they are “trigger-happy,” and much less susceptible to anergy. Clonal deletion of CD8+ T-cells also depends on Cbl-b, and Cbl-b-deficient T-cells are partially resistant to Treg cell-mediated suppression (83). Furthermore, induction of Tregs from naïve precursors is likewise impaired in the absence of Cbl-b (84).

The CD44+ memory phenotype T-cells generated by LIP are normally restrained by Tregs that proliferate rapidly in d3tx mice and are crucial for preventing autoimmunity in lymphopenic animals (50, 85). In *Aire*^−/−^/*Cbl-b*^−/−^ mice, readier activation of homeostatically proliferating T-cells, impaired induction of peripheral Tregs and lower responsiveness of proliferating lymphocytes to the influence of Tregs are probably responsible for their severe early autoimmunity. The proportions of CD4+ and CD8+ T-cells with CD44^high^ were greatly increased in these double knock-outs. This supports the idea that LIP is participating during prolonged lymphopenia in *Aire*^−^*^/^*^−^mice, where “trigger-happy” polyclonal T-cells proliferate in response to available self-peptide-MHC complexes in the presence of homeostatic cytokines.

Interestingly, the immune defects in NOD mice include mild lymphopenia and dysregulated function of homeostatic cytokines (46). Indeed, T-cell transfer and CFA injection protect NOD mice against diabetes (46). The efficiency of their thymic selection has been a matter of controversy; recent data are in line with normal negative selection but impaired positive selection in NOD mice due to selective defects in the Erk1/2 signaling module downstream of TCR (86) that is important for T-cell survival and tuning of TCR responsiveness. In the periphery, anergy induction appears normal in NOD T-cells. Insulin-specific effector T-cells were generated in pancreatic lymph nodes only between 3 and 5 weeks of age, at the time of increased release of β-cell antigens (87). In all mouse strains, a wave of β-cell apoptosis occurs during the neonatal period, peaking at 9–15 days, but apoptotic debris is cleared less efficiently in NOD mice (88). Interestingly, diabetes is accelerated in mice thymectomized at week 3 – i.e., precisely when β-cell-specific T-cells are initially activated – when Tx caused moderate lymphopenia. Furthermore, the timing of that lymphopenia is evidently critical in target organ selection; while d3tx in NOD mice did not affect diabetes incidence, gastritis became much commoner (88). Indeed, this *Aire*^−/−^/NOD combination may maximize homeostatic proliferation just when exocrine pancreatic antigen release is greatest. The combination of impaired positive selection in NOD mice with delayed migration of thymocytes into the Aire^−/−^ medulla apparently amplifies the neonatal lymphopenia, which is further exaggerated by hyper-responsiveness of NOD T-cells to IL-21 and poor T-cell survival. Homeostatically proliferating cells compete for IL-7 and/or available MHC/(cross-reactive) self peptides (56). Therefore the absence of diabetes in *Aire*^−/−^/NOD mice may implicate the early proliferation of T-cells that encounter other available autoantigens and fill the space before the β-cell antigens are released.

Why the autoimmune attack focuses on the exocrine pancreas remains obscure. We suggest that three peculiarities of neonatal mice might be relevant: (1) readier access of neonatal T-cells to peripheral organs (89) where they normally differentiate into TSA-specific Tregs (45). Interestingly, this conversion to Tregs is subverted by IL-7 (45); (2) rapid changes and increased blood flow to certain organs (lungs, pancreas, liver, and intestine) after birth that renders their antigens more accessible to T-cells; (3) autophagy that is naturally upregulated immediately after birth – to adapt to the loss of the constant trans-placental supply of nutrients – especially in muscle/diaphragm, heart and lungs; also the pancreas, which undergoes major changes after birth too, to meet the demands for the proteolytic enzymes it must now secrete (90). Their premature intracellular activation in autophagolysosomes, together with autoimmune attack by “trigger-happy” homeostatically proliferating T-cells, might greatly exacerbate the tissue damage. The thymic involution in *Aire*^−/−^/*Cbl-b*^−/−^ mice could be the result of stress or a “cytokine storm” created by this fulminant pancreatic disease.

## Treg Cells in Aire-Deficiency

Studies in APECED patients have shown significantly lower Treg numbers and function than in healthy controls (91–94). Whether this is a direct effect of the thymic AIRE-deficiency or secondary to the severe autoimmune diseases in these patients remains unknown. By contrast, the role of Aire-deficiency in the development of Treg cells in the mouse thymus is controversial. Many studies have reported that their numbers are unchanged (9, 18, 26, 95), but others have found them reduced (22, 96, 97). In peripheral organs, their numbers and function are similar to those in wt mice (9, 22). Recently, Malchow et al. showed appearance of Tregs specific for an Aire-dependent TSA that proliferated in tumors and could therefore interfere in their rejection (96). The autoimmunity in d3tx mice was initially thought to arise because of significantly later maturation and release of Tregs than of effector cells (55). However, Tregs proliferate equally well in d3tx lymphopenic hosts, which is important in the prevention of autoimmunity (50, 64). Interestingly, LIP is even greater in Tregs from *Aire*^−/−^ than wt mice when transferred to lymphopenic hosts (69).

One of the crosses that showed no additive effect on the phenotype of *Aire*^−/−^ mice was with *Card11^unm/unm^* (39). Normally, Card11 acts in the NFκB module of TCR-signaling, and this mutation leads to impaired Foxp3+ Treg differentiation in the thymus, 6–7 times fewer peripheral Tregs, and a gradual increase in Th2 cells (98). Interestingly, however, in *Aire*^−/−^ mice, these low-frequency Tregs could still reduce tissue infiltration. Furthermore, while Tregs are crucial for controlling autoimmunity against several organs, they seem to play no prominent role in eye disease: *FoxP3*-mutant scurfy mice do not develop spontaneous uveitis, suggesting that other tolerance mechanisms are more important than Tregs in protecting against retinal autoimmunity.

Also very informative are the crosses of B6.*Foxp3^sf^* mice (with the null “scurfy” *Foxp3* gene mutation) onto the *Aire*^−/−^ mice or NOD genetic backgrounds (99). The *Sf* mutation by itself causes characteristic skin disease, massive lymphoproliferation, and infiltration most severely in the liver, but also the lungs and exocrine pancreas (100, 101). The crosses onto both backgrounds started to develop more severe lung and liver infiltrates much earlier and died significantly younger than B6.*Foxp3^sf^* mice (99). While there were no changes in the infiltrates characteristically seen in other organs in B6.*Foxp3^sf^* mice, those typical of *Aire*^−/−^ mice on the C57BL/6 background (in the eyes, salivary glands) were – surprisingly – not seen in the B6.*Foxp3^sf^* Aire-deficient mice. Moreover, phenotypes were identical in *sf* mutant mice on these *Aire*^−/−^ and NOD backgrounds; to us, that implicates prolonged neonatal LIP rather than deficiency in thymic negative selection in this aggravated pathology in both crosses. *Sf* mutant Tregs are evidently not able to limit the activation of homeostatically proliferating T-cells. This is also illustrated by the similar wasting disease (with infiltrates in lungs, liver, pancreas, and stomach) in a model where neonatal T-cells are unable to respond to TGF-β signaling (102).

## What is Triggering Autoimmunity in APECED Patients?

If the mild phenotypes in *Aire*^−/−^ mice are in line with the requirements for pathogenic T-cell activation, why are the phenotypes so much more severe in APECED patients? In humans too, it seems very unlikely that defective negative selection is the only cause of the severe autoimmune destruction of endocrine glands and other tissues (6, 21, 103). We are born with a much more mature immune system than mice (104, 105). Although lymphocyte function is under-developed in neonates, their numbers per milliliters of blood are even higher than in adult humans. Therefore, even if thymocyte migration is delayed because of impaired chemokine secretion by AIRE-deficient mTECs in the human fetus, this is probably compensated by the longer gestation. Neonatal lymphopenia has not been studied in APECED because the disease is usually diagnosed much later. Interestingly though, adult APECED patients have increased IL-7 concentrations in their sera that may be related to impaired T-cell homeostasis (106). The clear differences in disease phenotypes between APECED patients and *Aire*^−/−^ mice suggest separate precipitating factor(s) in humans. These remain unidentified, but the surprisingly similar autoantibodies in patients with APECED and thymoma make any contribution from lymphopenia in human AIRE-deficiency seem even less likely (107). Nevertheless, the same logic – that additional activation is required before the rare naïve auto-reactive cells that escape from human AIRE-deficient thymi/thymomas can induce autoimmune disease – must apply in humans too (6, 103). In APECED, CMC, hypoparathyroidism, and Addison’s disease sometimes present even at 2–3 years of age (4). Evidently, T-cells must go onto attack very soon after birth to destroy sufficient tissue to cause disease so soon; to us, that argues against any need for environmental triggers. Moreover, the first targets of the autoimmune attack are not AIRE-dependent TSAs (21). We propose that the pathogenic T-cells are already primed before their export from AIRE-deficient thymi or thymomas. A study on T-cells in APECED adults has shown gross alterations, especially in the CD8+ population, that include increased proliferation, lower expression of both IL-7R and the negative regulator of TCR-signaling CD5, and also absence of the regular naïve T-cell compartment, relative to age-matched healthy controls (106). That could be secondary to the autoimmune diseases in APECED, a possibility that could be tested by assessing the activation of recent thymic emigrants before onset of APECED in pre-symptomatic young siblings of known patients.

In APECED, autoantibodies neutralizing type I IFNs and IL-22 can reach high titers even by 7 months of age, when autoantibodies to steroidogenic enzymes may also start to appear (108). Moreover, these autoantigens are produced in the thymus by cell types other than mTECs, so they should be available for negative selection even when AIRE is deficient (103). To explain these peculiarities, we have suggested biased selection or active autoimmunization in human thymi rendered “dangerous” by AIRE-deficiency (21, 103). That even leads to other secondary lymphoid tissue behavior in thymomas such as spontaneous production of anti-IFN-α and IL-12 autoantibodies by terminal plasma cells in sero-positive patients (109).

## Further Predictions

If gastritis in BALBc mice and EAU in B10.RIII mice are caused by LIP, they should be ameliorated by blocking homeostatic cytokines postnatally and simultaneously transferring lymphocytes into the lymphopenic hosts. As these cytokines sensitize TCRs through induction of pERK1/2, its inhibitors could be tested instead (65).

The phenotype of Cbl-b- and Aire double deficient mice could be mimicked by crossing with other mutant mouse strains with impaired T-cell susceptibility to anergy induction, or by thymectomizing *Cbl-b*^−/−^ mice on days 1–3.

Curiously, autoimmunity is more often related to lower than higher TCR-signaling, perhaps because of weaker peripheral tolerance (65, 86). During their development, cortical thymocytes are positively selected when their receptors are triggered by self-peptide-MHC complexes. These so called “tonic” signals are also needed for T-cell survival in the periphery, but they are regulated to remain just below the threshold for activation and proliferation (62). When TCR-signaling is impaired, the cells have to adapt to respond to weaker signals, which makes them more responsive to self-antigens, e.g., during periods of over-production of homeostatic cytokines. Theoretically, crosses of *Aire*^−/−^ mice onto backgrounds with decreased TCR-signaling and reduced T-cell survival could lead to phenotypes similar to those in *Aire*^−/−^ × NOD crosses.

## Summary

It is unlikely that defective negative selection of auto-reactive thymocytes in AIRE-deficient thymi is the only cause of the associated autoimmune diseases in either model mice or APECED patients. Naïve T-cells require activation before they can cause tissue destruction: in uninfected neonates with no danger signals, tolerization by peripheral mechanisms seems a much likelier outcome. A hitherto under-recognized feature of *Aire*^−/−^ mice is their prolonged neonatal lymphopenia: by inducing LIP, it favors the proliferation and activation particularly of auto-reactive T-cells. This also helps to explain the strikingly similar phenotypes of lymphopenic day 3 thymectomized and *Aire*^−/−^ mice. However, the many developmental (ontogenetic) differences make LIP seem a much less likely contributor in humans – where we propose that additional mechanisms promote the early and much more sharply focused autoimmune attack on such unusual targets as the parathyroids, steroidogenic tissues/enzymes, and cytokines.

The mouse model has been extremely valuable in demonstrating Aire’s role in negative selection of auto-reactive thymocytes. However, the differences in pathogenetic mechanisms and in autoimmune phenotypes in APECED patients question its suitability for testing new treatment options, and imply that merely restoring thymic TSA expression might not be enough to halt the autoimmunity in the patients. They also emphasize the importance of studies in human subjects, and again underline the need for caution when extrapolating from mouse models.

## Conflict of Interest Statement

The authors declare that the research was conducted in the absence of any commercial or financial relationships that could be construed as a potential conflict of interest.
